# A Chip Off the Old Block: Parents’ Subtle Ethnic Prejudice Predicts Children’s Implicit Prejudice

**DOI:** 10.3389/fpsyg.2018.00110

**Published:** 2018-02-09

**Authors:** Sabine Pirchio, Ylenia Passiatore, Angelo Panno, Fridanna Maricchiolo, Giuseppe Carrus

**Affiliations:** ^1^Department of Dynamic and Clinical Psychology, Sapienza University of Rome, Rome, Italy; ^2^Department of Education, Roma Tre University, Rome, Italy

**Keywords:** children’s explicit and implicit prejudice, blatant and subtle prejudice, parenting styles, child-Implicit Association Test, prejudice intergenerational transmission

## Abstract

The increasing flow of immigrants in many European countries and the growing presence of children from immigrant families in schools makes it relevant to study the development of prejudice in children. Parents play an important role in shaping children’s values and their attitudes toward members of other ethnic groups; an intergenerational transmission of prejudice has been found in a number of studies targeting adolescents. The present study aims to investigate the intergenerational transmission of ethnic prejudice in 3- to 9- year-old children and its relations to parenting styles. Parents’ blatant and subtle ethnic prejudice and parenting style are measured together with children’s explicit and implicit ethnic prejudice in pupils and parents of preschool and primary schools in the region of Rome, Italy (*N* = 318). Results show that parents’ subtle prejudice predicts children’s implicit prejudice regardless of the parenting style. Findings indicate that children might acquire prejudice by means of the parents’ implicit cognition and automatic behavior and educational actions. Implications for future studies and insights for possible applied interventions are discussed.

## Introduction

The multiethnic character of increasingly large parts of urban and rural communities in western societies makes the topic of interethnic contact more and more relevant in order to improve the quality of life and personal relations in daily life contexts, and to foster social inclusion. Prejudice reduction in adults and children is a crucial issue in this process (Passiatore et al., 2017). The dynamics of prejudice formation and expression in adults was investigated quite in depth in the field of social psychology and social sciences in general (Aboud and Steele, 2017). While the presence of ethnic prejudice is manifest in children as young as 3 years old in terms of ingroup favoritism and outgroup discrimination (Dweck, 2009), the identification of the environmental sources of ethnic prejudice in children still needs to be studied.

Long before they are able to identify themselves in relation to a social group, infants in their first year of life show early capacities of social discrimination such as a clearer preference for faces of their same-ethnicity (Kelly et al., 2007), for someone speaking their language and for toys selected by someone speaking their own language (Kinzler et al., 2007). The same pattern of results can be observed regarding the children’s preference for familiar people and social stimuli. An ethnicity bias clearly emerges in children as young as 3 or 4 years of age (Aboud, 1988; Augoustinos and Rosewarne, 2001; Bigler and Liben, 2006; Aboud, 2008; Gaither et al., 2014), it appears to reach its peak in middle childhood around 7–8 years old, and then it gradually declines. This trend does not apply to all types of children’s racial or ethnic attitudes, differentiating instead between explicit and implicit attitudes: the former are conscious and under intentional control while the latter are automatic and relatively out of attentional control (see for example, Dunham et al., 2013; Baron, 2015). These attitudes do not correlate with each other: on the one hand, explicit attitudes grow until middle childhood and then progressively decline; on the other hand, implicit ethnic attitudes seem to be relatively stable over the time (e.g., Rutland, 1999; Degner and Wentura, 2010). For example, 6 years old children are equally biased for both implicit and explicit levels (Baron and Banaji, 2006), while 10 years old children show a dissociation between the two measures (i.e., the level of implicit ethnic attitudes stays constant while self-reported preferences for the own ingroup decrease). This process is likely due to the increased effect of social norms prohibiting the explicit expression of negative ethnic attitudes. Such social norms might inhibit the overt expression of these attitudes without reducing the bias that becomes observable only in implicit tasks. The activation of automatic prejudice and the expression of explicit prejudice not only follow different developmental paths but also use two distinct modes of processing (Moors and De Houwer, 2006). Broadly speaking, automatic processes operate regardless of attentional resources and they do not require intention as opposed to the non-automatic processes that, differently, are subject to the person’s awareness and require more attention and cognitive effort. Measures of explicit attitudes reflect deliberate and controlled responses that can be altered or suppressed, whereas implicit measures tap automatic and spontaneous processes that are difficult to consciously modify or extinguish (Schneider and Shiffrin, 1977). Associative processes give rise to implicit attitudes independently from subjective truth or falsity (Gawronski and Bodenhausen, 2006), and in turn, they may predict discriminatory behaviors (Gawronski and Bodenhausen, 2007), and affect social judgments and interpersonal relations (Dovidio et al., 2002). Therefore, the development and expression of implicit ethnic attitudes in children is a worth issue for developmental and educational studies. In particular it is relevant to better clarify the role of different socializing agents, such as parents and the education system, in the development and transmission of prejudice in childhood. Based on these assumptions, the goal of this study consists of investigating the expression of children explicit and implicit prejudice and the prejudice transmission process from parents to children.

### The Transmission of Ethnic Attitudes and Prejudice

The development of intergroup attitudes is conceivable as the result of the interaction between genetic predispositions, socialization influences, and situational determinants (Hatemi et al., 2009; Verhust and Hatemi, 2013). Children’s attitudes crucially depend on early socialization experiences and therefore are influenced by significant adults (Carlson and Iovini, 1985; Bigler and Liben, 2007; McGlothlin and Killen, 2010). Socializing agents play a role in this process, suggesting children’s intergroup attitudes to be a function of the attitudes of their parents through the process of social transmission (Radke-Yarrow et al., 1952; Allport, 1954; Bandura, 1977; Aboud and Amato, 2001; Nesdale and Flesser, 2001). Regarding ethnic prejudice, a number of researches confirm a strong similarity between the parents’ and their children’s racial attitudes (O’Bryan et al., 2004; Jaspers et al., 2008; Rodriguez-Garcia and Wagner, 2009; Dhont and Van Hiel, 2012; Dhont et al., 2013; Meeusen, 2014), while other studies find only a limited correspondence (Aboud and Doyle, 1996; Hello et al., 2004; Vittrup and Holden, 2011). A recent meta-analysis highlights that children’s and parents’ intergroup attitudes are related with each other, showing ranges from small to moderate effect sizes (Degner and Dalege, 2013). According to this meta-analysis, a relevant factor in such effect sizes’ variability is the direct vs. indirect measurement of parent’s prejudice, as indirect assessments (e.g., asking children to assess their parent’s prejudice) might lead to inflated estimates of children–parent correlations. Thus, in our study, we relied only on direct assessments of both children and parents prejudice.

Furthermore, intergenerational transmission may be stronger when the significant adults engender a close relationship with their children (Sinclair et al., 2005b) or when children are highly identified with their significant adults (Sinclair et al., 2005a). These findings lead to some considerations about the nature of the mechanisms regulating intergenerational transmission. Studies on the transmission of values between parents and adolescents indicate that the age of the child and the content of the values or attitudes may play a role, together with the authoritativeness of the parents’ educational style (e.g., Pinquart and Silbereisen, 2004). If the role of parents, and of the quality of the parent-child relationship, for children development and adjustment is unequivocally clear (Stevenson et al., 1990; Rodriguez-Garcia and Wagner, 2009), on the other hand, the relationship between specific parenting styles and the child’s outcomes in terms of competence, self-confidence and emotional control are controversial. Some studies indicate the authoritative parenting style as the best for children well-being and achievement (Pinquart, 2016, 2017) and others collect evidences of a more multifaceted phenomenon where the children’s outcomes cannot be predicted by a specific parenting style (García and Gracia, 2009).

Therefore, it is interesting to clarify the relationship between parenting style and prejudice transmission. It has been suggested that parenting variables, such as the perceived support, affect the parents’ prejudice influence on children attitudes (Miklikowska, 2016). Some studies considered individual factors such as, for instance, authoritarianism, social dominance, intrinsic *vs.* extrinsic goals, or external vs. internal orientation in prejudice formation and expression (e.g., Duriez et al., 2008; Sibley and Duckitt, 2008; Duriez, 2011). However, studies explicitly considering the effect of specific parenting style on children ethnic prejudice and including a direct measure of parenting style are still lacking, and would shed light on the role of educational practices on prejudice development.

Thus, in the present study we measured the influence of parenting style construct articulated in three different forms, authoritative, authoritarian, permissive on the relationship between parents and children ethnic prejudice.

Another important aspect that did not received sufficient consideration in previous studies on prejudice’s transmission is the distinction between explicit or blatant and implicit or latent forms of prejudice. It is arguable that both can be influenced by parents’ attitudes and practices in different ways.

At the explicit level, children tend to imitate and conform to the explicit attitudes and behaviors of their parents. Also for implicit ethnic attitudes, a number of studies report a similarity between children and adults’ patterns of prejudice (e.g., Sinclair et al., 2005a; Dunham et al., 2006; Castelli et al., 2009; Vezzali et al., 2012). These findings, however, are related mostly to adolescent or children above the age of 10. The finding that implicit attitudes are acquired within the family and that they are probably linked to early socialization experiences, needs to be confirmed in relation to preschool or primary school years. Furthermore, it is still not clear if parenting practices are responsible for the transmission of attitudes, or if children conform to their parents’ behaviors, and which is the role of the other environmental factors in this process.

### The Present Study

The general purpose of the current study is to fill in the existing gaps in the previous literature on internal and family-agents influencing the explicit and implicit ethnic prejudice transmission in children, by expanding and generalizing the findings of previous studies.

A previous work by Castelli et al. (2009) found for example that implicit prejudices of mothers, predicts the racial attitudes of their preschool children; these authors however, only use an explicit measure. Thus, in the present study, we assess children’s implicit ethnic attitudes through an implicit measure, such as the Implicit Association Test.

Other few studies analyzed the intergenerational transmission of implicit attitudes from parents to their children but they only include pre-adolescents (e.g., Sinclair et al., 2005a). Thus, in the present study, we consider preschool and primary school children (3 to 9 years old).

Also, as previously mentioned, we want to take into account also the role of parenting styles on the ethnic prejudice transmission, which was underinvestigated by previous studies.

In sum, our study aims to analyze: (a) the role of the parents’ blatant and subtle prejudice on their children explicit and implicit prejudice; (b) the role of parenting styles in the prejudice transmission from parents to children; (c) the role of children age in the development and expression of children explicit and implicit ethnic prejudice. Coherently with all the literature reviewed so far (e.g., Baron, 2015), we expect that:

(1) the parents’ subtle ethnic prejudice would predict the child’s implicit prejudice, also after controlling for the parenting style (H1);
(2) the parents’ blatant ethnic prejudice would correlate with the child’s explicit ethnic prejudice (H2);
(3) significant differences in children prejudice across age groups would be present only in the case of explicit prejudice (H3).

## Materials and Methods

### Participants

A total of 318 children and one parent for each of them participated to the study, in the context of a larger EU-funded research and intervention project (The *SOFT-School and family together for the integration of immigrant children* project, funded in 2012–2015 by the LLP-KA2MP program). The study protocol was approved by the directors of the involved schools. Parents were asked to voluntary participate to the study and expressed their informed consent to the involvement of their children in the study. The children attended 11 preschool and primary schools in the cities of Rome and Fondi, Latium region, Italy. They are all Caucasian children with Italian nationality and ranged in age from 3 to 9 years (*M* = 6.06; *SD* = 1.86). Girls constituted 46% of the sample. The involved schools were located in neighborhoods with similar socio economic conditions (e.g., low-middle class), and the involved classrooms were characterized for similar proportions of pupils with an immigrant background.

### Procedure and Instruments

#### Parents

Parents received at home the questionnaire and one parent per family completed it. All questionnaires were completed by mothers. The questionnaire included two scales:

1. the Italian adaptation (Arcuri and Boca, 1996) of the questionnaire by Pettigrew and Meertens (1995). The scale is composed of 20 items measuring ethnic prejudice on a five-point Likert scale (1 = totally disagree; 5 = totally agree). Ten items measure the blatant prejudice (e.g., “Immigrants take jobs that should be up to Italians”) and 10 measure the subtle prejudice (e.g., “Immigrants that live in our country transmit to their children values and abilities not required in Italy”). The total prejudice is the sum of blatant and subtle prejudice. High scores correspond to high prejudice levels.
2. Parenting was measured by a 32 items version of the *“Parent Style and Dimension Questionnaire”* (Robinson et al., 2001) that describes three parenting profiles: authoritative (e.g., “I am responsive to my child’s feelings and needs”), authoritarian (e.g., “I use criticism to make my child improve his/her behavior”), and permissive (e.g., “I find it difficult to discipline my child”). The scale was back translated in Italian. Scores range on a five-point scale (1 = never; 5 = always).

#### Children

Children were tested individually at school. For measuring their explicit ethnic prejudice, they were shown six photos of children (six boys or six girls according to the gender of the participant child) selected from six different ethnic groups (Caucasian, African, Asiatic Indians, Asiatic Chinese, Arabian, South-American; Lo Coco et al., 2000). The ethnic groups were chosen according to the composition of immigrant population in the area of the research. The six photos were positioned in front of the child who are required to indicate: (1) the child that he/she would like to be; (2) the two children that he/she would like to invite at home to spend some time together. Next the experimenter asked to the child to assign adjectives and verbs written on a card and read by the experimenter to each child: seven positive adjectives (nice; good; clever; clean; likeable; happy; obedient), seven negative adjectives (bad, nasty, stupid, dirty, unpleasant, unhappy, disobedient), three positive verbs (give, hug, help), three negative verbs (steal, beat, disturb), and three neutral verbs (watch the TV, eat, sleep). We assigned score “1” when the student chose a child who belong to his/her ethnicity. We assigned “0” when the student chose a child who doesn’t belong to his/her ethnicity. The global score of explicit ethnic prejudice is composed by the sum of scores at items 1 and 2 and the score at the positive and negative adjectives and verbs (the score “0” of negative adjectives and verbs was converted in “1”). Therefore, the total score in this variable could range from 0 (low explicit prejudice) to 22 (high explicit prejudice).

Children’s implicit ethnic prejudice was assessed by the child version of the IAT (Child-IAT) adapted to comply with the recommendations by Baron and Banaji (2006) and designed using Super Lab Pro 4. The test consisted of seven blocks (Nosek et al., 2005). In the first block the students were asked to categorize photographs of Caucasian, African, Asiatic Indians, Asiatic Chinese, Arabian, South-American children (the photographs employed are different from explicit prejudice test) in two different groups – Caucasian and Other – represented by two pictures (Caucasian children group and Multi-ethnic group). In the second block, they categorized verbal stimuli by positive (nice, good, happy, boring, clear) or negative valence (bad, nasty, unhappy, funny, dirty) in two different groups represented by two faces: a green and happy face for the positive stimuli and a red and unhappy face for negative stimuli. The verbal stimuli are audio-recorded to allow pre-school children to participate and to avoid interferences of the different reading abilities of the primary school children with the test’s performance. The next two blocks were combined categorization tasks: respondents were asked to press the left (blue) button when a stimulus in either the “Caucasian” category or “Positive” category appeared and the right (yellow) button when a stimulus in the “Other” or “Bad” category appeared. The third block was a practice block while the fourth block was “critical” block. The fifth block consisted of another simple categorization task, however, with the reversed positioning of “Caucasian” and “Other.” This was done to avoid the influence of previously learned spatial positions of category names (Schnabel et al., 2007). Blocks 6 and 7 were analogous to blocks 3 and 4, with the opposite pairing of the target and attribute categories. Each stimulus was presented until the child provided a response, after which an inter-stimuli period of 150 ms followed. Incorrect answers were followed by red symbol “X” in the center of the screen. The child can’t move on until the correct answer is given. The data were filtered excluding those with latency higher than 10000 ms and with correct answers’ mean inferior to 0.80. The wrong answers were replaced by correct answers’ mean penalized with 600 ms. The Cronbach Alpha is 0.84.

### Statistical Analysis

To investigate the relationships involving parents’ ethnic prejudice and children’s ethnic prejudice, we computed zero-order correlations among these variables (the 0.05 level of significance was adopted throughout all analyses). Descriptive statistics of our sample are also provided. To test our main hypotheses (H1 and H2), we carried out two multiple regressions, in which parenting styles and parents’ ethnic prejudice (blatant and subtle) were entered as predictors of children’s implicit and explicit ethnic prejudice, respectively. To test our hypothesis on the different effects of age on explicit vs. implicit prejudice (H3), we created three different age groups (according to previous literature on ethnic prejudice development, e.g., Raabe and Beelmann, 2011): preschool (3–5 years old); first class of Primary school (6–7 years old); fourth class of Primary school (9 years old). Then we tested age-group differences in explicit and implicit prejudice through two univariate ANOVAs.

## Results

Descriptive statistics and bivariate correlations linking parents’ and children’s ethnic prejudice measures are displayed in **Table 1**.

**Table 1:** Means, standard deviations, bivariate correlations, and Cohen’s *d*.

	1	2	3	4	5	6	7
(1) Parents Blatant	-						
(2) Parents Subtle	*r* = **0.57**	-					
	*p* = 0.000						
	*d* = 1.38						
	*n* = 249						
(3) Child-Implicit	*r* = 0.06	*r* = **0.18**	-				
	*p* = 0.495	*p* = 0.033					
	*d* = 0.12	*d* = 0.37					
	*n* = 144	*n* = 145					
(4) Child-Explicit	*r* = -0.01	*r* = 0.00	*r* = -0.02	-			
	*p* = 0.858	*p* = 0.978	*p* = 0.71				
	*d* = -0.02	*d* = 0.00	*d* = -0.04				
	*n* = 204	*n* = 205	*n* = 175				
(5) Authoritative	*r* = **-0.27**	*r* = -0.11	*r* = -0.11	*r* = 0.06	-		
	*p* = 0.000	*p* = 0.088	*p* = 0.088	*p* = 0.331			
	*d* = -0.56	*d* = -0.22	*d* = -0.22	*d* = 0.12			
	*n* = 249	*n* = 250	*n* = 250	*n* = 277			
(6) Authoritarian	*r* = **0.26**	*r* = **0.18**	*r* = 0.01	*r* = -0.09	*r* = **-0.35**	-	
	*p* = 0.000	*p* = 0.005	*p* = 0.913	*p* = 0.156	*p* = 0.000		
	*d* = 0.54	*d* = 0.37	*d* = 0.02	*d* = -0.18	*d* = -0.75		
	*n* = 249	*n* = 250	*n* = 152	*n* = 277	*n* = 361		
(7) Permissive	*r* = **0.15**	*r* = 0.12	*r* = -0.021	*r* = 0.03	*r* = -0.09	*r* = **0.42**	-
	*p* = 0.000	*p* = 0.071	*p* = 0.797	*p* = 0.59	*p* = 0.076	*p* = 0.000	
	*d* = 0.30	*d* = 0.24	*d* = -0.04	*d* = 0.06	*d* = -0.18	*d* = 0.93	
	*n* = 248	*n* = 249	*n* = 151	*n* = 276	*n* = 360	*n* = 360	
*M* (*SD*)	2.43 (0.59)	2.90 (0.51)	0.19 (0.38)	12.30 (2.59)	4.07 (0.51)	1.98 (0.62)	

*Missing values are deleted listwise. Parents Blatant = parents’ blatant prejudice; Parents Subtle = parents’ subtle prejudice; Child-Implicit = children’s explicit prejudice; Child-Explicit = children’s explicit prejudice; Authoritative = parents’ authoritative style; Authoritarian = parents’ authoritarian style; Permissive = parents’ permissive style. The bold values indicate significant correlations.*

As predicted, children’s implicit ethnic prejudice is significantly and positively related to parents’ subtle prejudice. No significant correlation emerges between parents’ blatant prejudice and children either explicit or implicit prejudice. Concerning parenting styles, no relations emerged with children’s prejudice. However, as arguable, parent’s prejudice (especially blatant prejudice) is positively correlated with an authoritarian parenting style, and negatively correlated with an authoritative parenting style. Also, interestingly, children’s implicit and explicit prejudice are not significantly associated each other, in line with previous literature. On the contrary, parent’s blatant and subtle prejudice scores are significantly and positively correlated.

In line with our main hypothesis (H1), a multiple regression analysis showed that parents’ subtle prejudice has a significant positive effect on children’s implicit ethnic prejudice (β = 0.21; *p* = 0.04), also after controlling for parenting styles and parent’s blatant prejudice. No significant effects of parenting styles and parent’s blatant prejudice on children implicit ethnic prejudice were detected (*p* > 0.1).

**FIGURE 1:**
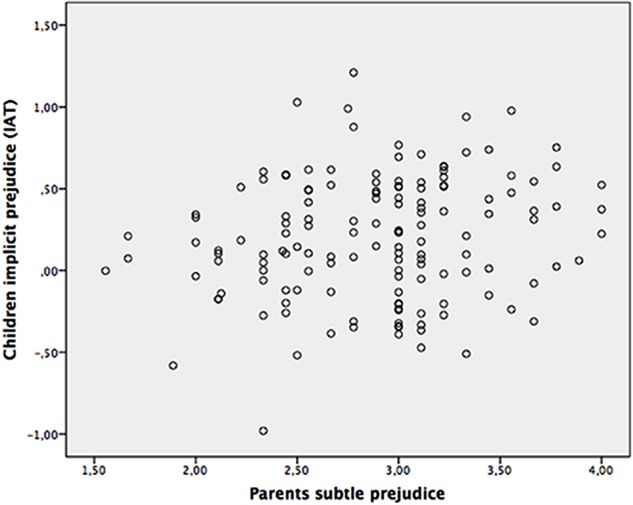
Scatterplot of relations between parents subtle prejudice and children implicit prejudice (IAT).

**FIGURE 2:**
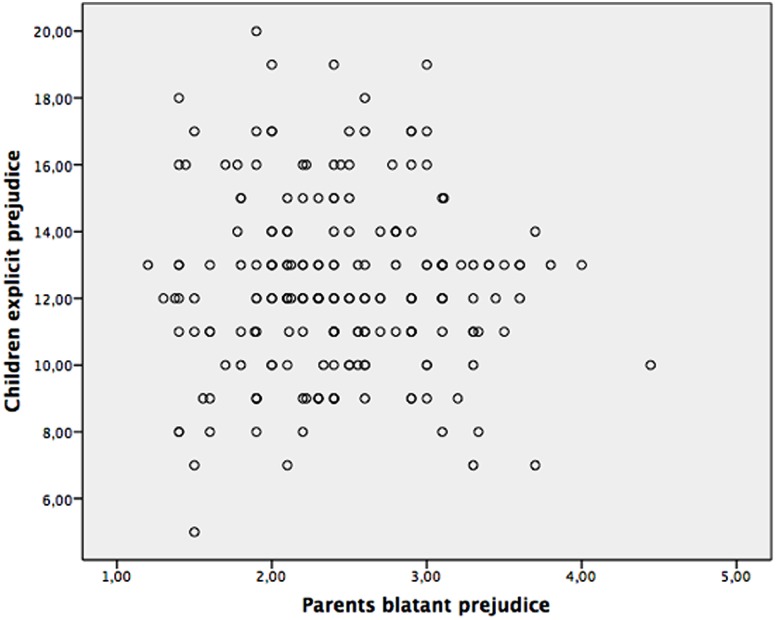
Scatterplot of relations between parents blatant prejudice and children explicit prejudice.

The multiple regression conducted testing our second hypothesis showed no significant effects of parents’ blatant and subtle prejudice and parenting styles on children’s explicit prejudice. Scatterplot graphs for the parent-child prejudice links were also run (subtle-implicit and blatant-explicit, respectively); these graphs are displayed in **Figures 1**, **2**, and show that the significant and non-significant effects we detected are not likely to be attributed to a few outliers.

Children’s prejudice scores across the different age groups are showed in **Table 2**. As predicted in our second hypothesis, univariate ANOVAs confirms the significant variation of explicit ethnic prejudice across age (*F*_(2,341)_ = 4.13, *p* = 0.017). The ethnic explicit prejudice significantly increases from preschool age (3–5 years) to middle childhood (6–7 years) followed by a decrease in the late childhood (9 years). Duncan *post hoc* comparison revealed indeed that explicit prejudice scores in 6–7 years old children were significantly higher compared to both 3–5 and 9 years old children (*p* < 0.05); these, in turn, did not significantly differed each other (*p* = 0.96). Conversely, as expected, no significant variations of implicit prejudice emerged across the three age groups (*F*_(2,198)_ = 0.686, *p* = 0.505).

**Table 2:** Means and standard deviations in children explicit and implicit ethnic prejudice across age groups.

	3–5 years		6–7 years		9 years	
	*M*	*SD*	*M*	*SD*	*M*	*SD*
Explicit prejudice	12.03^a^	2.55	12.90^b^	2.57	12.04^a^	2.60
Implicit prejudice	0.17^a^	0.47	0.16^a^	0.40	0.23^a^	0.30

*Means with different superscripts are different for *p* < 0.05.*

## Discussion

The purpose of the present study was to investigate the role of parental influences in the development and expression of ethnic prejudice in preschool and primary school children, in particular taking into account the distinction between different forms (i.e., explicit vs. implicit) of prejudice in adults and children.

While it is widely acknowledged that the social environment and relevant socialization agents (such as parents) certainly play a role in shaping relevant social attitudes in children and adolescents, researches so far have described the nature and entity of these relations in inconsistent ways. By the use of explicit and implicit prejudice measures in children (and blatant vs. subtle prejudice measures in their parents) we wanted to ascertain the process of intergenerational transmission of ethnic prejudice.

Our data are coherent with previous findings (e.g., Castelli et al., 2009) showing that parental blatant and subtle prejudice are differently linked to young children’s prejudice. In fact, while children’s explicit prejudice seems unrelated to their parents either blatant or subtle prejudice, it is not surprising that children’s implicit ethnic prejudice is positively predicted by parents’ level of subtle ethnic prejudice, given the extensive amount of empirical evidence on dual process accounts of human cognition, affect, decision making, and behavior (Bargh and Chartrand, 1999; Kahneman, 2002; Strack and Deutsch, 2004). However, our findings do not contradict the existing literature on the importance of imitation process in child–adult relations (e.g., Koffka, 1935; Piaget, 1946; Bandura, 1977). Our findings suggest that children’s prejudice may be rooted in the automatic behavior and implicit social influence processes enacted by their significant adults, more than in what parents explicitly think (and likely say) about ethnically different people to their children. This findings also strengthen the idea that implicit and explicit attitudes are activated and expressed through different channels and processes (Schneider and Shiffrin, 1977; Moors and De Houwer, 2006). Children’s explicit prejudice is under their intentional control (and therefore might be mitigated by social norms that discourage the overt expression of negative ethnic attitudes) more than under the influence of their parents’ blatant attitudes. On the contrary, the children’s implicit attitudes, being out of their intentional control, might be influenced by the subtle parents’ prejudice. In sum, the children’s life environment, including parent’s attitudes, do play a role, but the different facets of implicit and explicit social cognition should be considered to fully account for prejudice development in childhood (e.g., Baron and Banaji, 2006; Castelli et al., 2009).

Finally, we have not found clear evidence for a direct role of parenting styles in the intergenerational transmission of ethnic prejudice: the parents’ subtle prejudice levels have a role in predicting the children implicit prejudice independently of the specific parenting style put in practice by parents. Nevertheless, since our data show that an authoritarian parenting style is positively linked to parent’s prejudice (both blatant and subtle), while an authoritative style is negatively linked to parent’s blatant prejudice, it might well be that parenting styles exert an indirect influence on parental prejudice transmission, particularly at an implicit level.

The present findings also confirm our general assumption that implicit and explicit ethnic prejudice in children are distinct phenomena, and might follow different developmental patterns. In fact, implicit prejudice scores in our sample are relatively constant in children ranging from 3 to 9 years old while explicit prejudice in 6–7 years-old children show higher scores than in 3–5 and 9 years-old children.

Some limitations must however be acknowledged in our study. First, we measured implicit prejudice in parents by a self-report measure, such as the Pettigrew and Meertens questionnaire (Pettigrew, 1991; Pettigrew and Meertens, 1995; Meertens and Pettigrew, 1997). Although this scale is acknowledged as the most referential tool to measure the two types of prejudice, the use of self-report measures for implicit constructs may be questionable, since answering requires a certain degree of the respondents’ awareness about their attitude toward the item’s content (Hoffman et al., 2005). The fact that both explicit and implicit prejudice of the parents were measured with questionnaires (e.g., Castelli et al., 2009) might not allow to draw definitive conclusions about the true implicit prejudice of the parents. For example, is it possible that the highly correlated parents’ subtle and blatant prejudice scores are due to the fact that they were both measured with a questionnaire. Furthermore, it should be considered that the statements in parents’ questionnaires are about immigrants versus native Italians, while in children’s explicit prejudice measures children are asked to judge groups that differ not only on ethnicity, but also on language and culture, which is something more complex than just “us versus other.” Future studies, therefore, could extend and confirm our findings by means of actual implicit measures for both parents and children.

Also, the lack of influence of parenting on the intergenerational transmission of ethnic prejudice in our findings might be the effect of the instrument we used to measure parenting style. In fact, especially for the assessment of authoritarian parenting style, the formulation of the items could have discourage parents to provide reliable answers because of social desirability (e.g., “I spank my child when I don’t like what he/she does or says”).

Finally, it is worth to point out that the present findings might have relevance from an applied point of view. The role of parents in shaping the socialization experiences of their children is well-established in the framework of ecological development (Bronfenbrenner, 1979) and by studies on interethnic relationships in adolescents and children (Bifulco et al., 2009; Edmonds and Killen, 2009; Munniksmaa et al., 2012). Because of the increasing multiethnic character of daily life environments in many current human societies (Vertovec, 2007), interethnic relations and inclusiveness is an important issue for children and adolescent in schools (e.g., Thijs and Verkuyten, 2014; see also Graham and Cohen, 1997; Pirchio et al., 2015). It is widely acknowledged that the quality of interethnic relationships may contribute to academic achievement and well-being for both majority and minority groups students (Driessen, 2002; Van Ewijk and Sleegers, 2010; Thijs et al., 2014).

Knowing that the parents’ influence on children ethnic attitudes can follow also an implicit and automatic path may offer two important indications for educational and psychological intervention programs aiming at reducing ethnic prejudices in children, and therefore fostering positive interethnic relationships. The first one relates to the need of targeting not only children, but also parents for reducing the children’s prejudice. This might help to avoiding feelings of threat and incoherence in children who eventually would be faced with interventions that propose attitudes and values that are in contrast with parents’ explicit or implicit attitudes. The second one is related to the importance of addressing not only the explicit and aware side of ethnic prejudice, but also tackling the latent and implicit attitudes which the parents might not be aware of, but which are able of activating and reinforcing children’s prejudice (Patterson and Bigler, 2006). In this direction, interventions based on the Contact Hypothesis theory (Kawakami et al., 2007; Thijs and Verkuyten, 2014) and involving the children together with their significant adult (Pirchio et al., 2017a,b) could give positive outcomes.

## Conclusion

Our findings offer an important contribution to plan and design effective social and educational intervention programs for prejudice reduction involving parents as intervention targets for effective attitudes’ changes in children. Exploring the possible synergies between different individual and contextual factors at the basis of a more socially inclusive and sustainable human experience in both education and daily life contexts involving human relations to the surrounding environment (e.g., Carrus et al., 2015, 2017; Mercado-Domeìnech et al., 2017; Panno et al., 2017) is also a worthy issue for future research investigating the effectiveness of social inclusion interventions programs in schools.

## Ethics Statement

Adult participants to this study expressed their oral consent to take part to the survey, and voluntary accepted to fill in a self-reported paper and pencil questionnaire. For children participants, parents gave written informed consent to participation. This study did not require approval from an ethical committee according to Italian regulations.

## Author Contributions

SP and YP equally contributed to writing the text (shared first authorship); AP, GC, YP, and SP equally contributed to conducting the statistical analysis and revising the text; YP collected the data; FM contributed to implement the child IAT procedure (defining the script, data coding, and analyzing).

## Conflict of Interest Statement

The authors declare that the research was conducted in the absence of any commercial or financial relationships that could be construed as a potential conflict of interest.
